# Exploring protocol development: Implementing systematic contextual memory to enhance real-time fMRI neurofeedback

**DOI:** 10.2478/joeb-2024-0006

**Published:** 2024-05-31

**Authors:** Steffen Maude Fagerland, Henrik Røsholm Berntsen, Mats Fredriksen, Tor Endestad, Stavros Skouras, Mona Elisabeth Rootwelt-Revheim, Ragnhild Marie Undseth

**Affiliations:** 1 The Intervention Centre, Division of Technology and Innovation, Oslo University Hospital, Oslo, Norway; 2 Department of Cognitive and Neuropsychology, Department of Psychology, University of Oslo, Oslo, Norway; 3 RITMO Centre for Interdisciplinary Studies in Rhythm, Time and Motion, Department of Psychology, University of Oslo, Norway; 4 Neuropsychatric Outpatient Clinic, Vestfold Hospital Trust, Tønsberg, Norway; 5 Department of Neuropsychology, Helgeland Hospital, Norway; 6 Department of Fundamental Neurosciences, Faculty of Medicine, University of Geneva, Geneva, CH-1202, Switzerland; 7 Department of Biological and Medical Psychology, University of Bergen, Bergen, NO-5020, Norway; 8 Department of Neurology, Inselspital University Hospital Bern, Bern, CH-3010, Switzerland; 9 Institute of Clinical Medicine, Faculty of Medicine, University of Oslo, Oslo, Norway; 10 Division of Radiology Research, The Intervention Centre, Oslo University Hospital, Oslo, Norway

**Keywords:** rtfMRI-nf, ADHD, Tourette’s Syndrome, VR

## Abstract

**Objective:**

The goal of this study was to explore the development and implementation of a protocol for real-time fMRI neurofeedback (rtfMRI-nf) and to assess the potential for enhancing the selective brain activation using stimuli from Virtual Reality (VR). In this study we focused on two specific brain regions, supplementary motor area (SMA) and right inferior frontal gyrus (rIFG). Publications by other study groups have suggested impaired function in these specific brain regions in patients with the diagnoses Attention Deficit Hyperactivity Disorder (ADHD) and Tourette’s Syndrome (TS). This study explored the development of a protocol to investigate if attention and contextual memory may be used to systematically strengthen the procedure of rtfMRI-nf.

**Methods:**

We used open-science software and platforms for rtfMRI-nf and for developing a simulated repetition of the rtfMRI-nf brain training in VR. We conducted seven exploratory tests in which we updated the protocol at each step. During rtfMRI-nf, MRI images are analyzed live while a person is undergoing an MRI scan, and the results are simultaneously shown to the person in the MRI-scanner. By focusing the analysis on specific regions of the brain, this procedure can be used to help the person strengthen conscious control of these regions. The VR simulation of the same experience involved a walk through the hospital toward the MRI scanner where the training sessions were conducted, as well as a subsequent simulated repetition of the MRI training. The VR simulation was a 2D projection of the experience.

The seven exploratory tests involved 19 volunteers. Through this exploration, methods for aiming within the brain (e.g. masks/algorithms for coordinate-system control) and calculations for the analyses (e.g. calculations based on connectivity versus activity) were updated by the project team throughout the project. The final procedure involved three initial rounds of rtfMRI-nf for learning brain strategies. Then, the volunteers were provided with VR headsets and given instructions for one week of use. Afterward, a new session with three rounds of rtfMRI-nf was conducted.

**Results:**

Through our exploration of the indirect effect parameters – brain region activity (directed oxygenated blood flow), connectivity (degree of correlated activity in different regions), and neurofeedback score – the volunteers tended to increase activity in the reinforced brain regions through our seven tests. Updates of procedures and analyses were always conducted between pilots, and never within. The VR simulated repetition was tested in pilot 7, but the role of the VR contribution in this setting is unclear due to underpowered testing.

**Conclusion:**

This proof-of-concept protocol implies how rtfMRI-nf may be used to selectively train two brain regions (SMA and rIFG). The method may likely be adapted to train any given region in the brain, but readers are advised to update and adapt the procedure to experimental needs.

## 2. Introduction

Bio- and neuro-feedback relates to monitoring one’s own biological indicators with the aim of inducing enhanced conscious control of them. Feedback from sensors used to record these indicators (e.g. pulse, heartrate variability, bioimpedance, fMRI, fNIRS) may be presented to the individual using a watch, mobile phone, computer screen or VR googles. Neurofeedback based on fMRI allows monitoring of own brain activity. Conscious and repeated activation of specific regions of the brain may help establish new circuits, which may potentially strengthen these regions. Outcomes after neurofeedback training can be measured using activity measurements (degree of directed stream of oxygenated hemoglobin toward the area) and connectivity (degree of correlated activity in separate regions), and these measures may be correlated with validated questionnaires for clinical outcomes.

Several sensors have been developed to monitor changes in, for example, heart rate, skin conductance, or muscle tone, reflecting arousal. Indicators of brain activity can be monitored through, for example, electroencephalogram (EEG) or functional Magnetic Resonance Imaging (fMRI) [5, 6, 7, 8, 9]. Previous studies on biofeedback have found that approaches aimed at gaining control over one’s own physiology may have therapeutic potential for conditions such as epilepsy, ADHD, chronic pain, depression, and anxiety [9, 10, 11, 12, 13].

**Figure 1:**
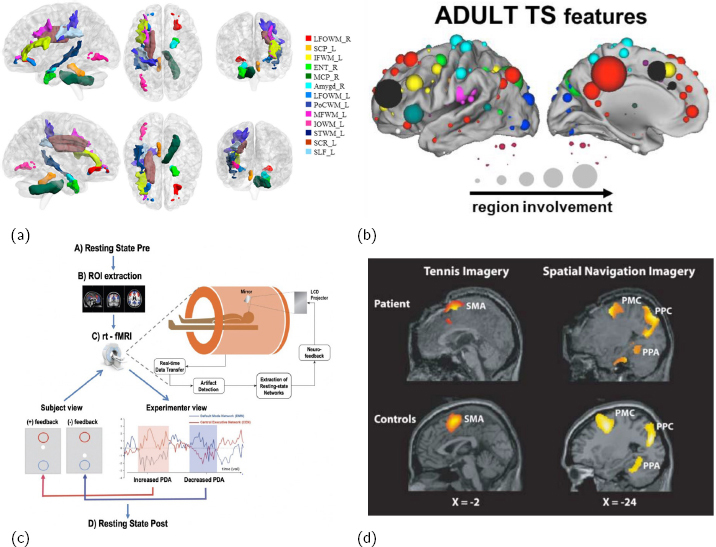
Discovered deviations in the brain of patients with TS mapped through (a) diffusion tensor imaging (DTI) and (b) connectivity; (c) a schematic of the setup of rtfMRI-nf, showing an overview of the required instruments and sequence of events; (d) when comparing the strategic thinking of assumed vegetative patients with that of healthy controls, it was discovered that these patients were, in fact, not vegetative. Figures (a,b,c,d) from ([1],[2],[3],[4]), respectively.

Improved biofeedback methods may have widespread applicability in selectively inducing new brain circuits, and may thus potentially be used for correcting impairments or disorders affecting cognitive functions. The approach we explored the development of in this publication aimed to allow repeating fMRI brain training at home. Recent studies using machine learning were able to distinguish patients with Tourette’s Syndrome ([1, 2], Fig. 1a,b) and ADHD ([14]) from healthy controls, implying that structural and functional alterations may be a part of the origin of these diagnoses. Therefore, the ability to consciously tune such alterations, reducing the deviation from healthy controls, may reduce the severity of such conditions.

Real-time fMRI neurofeedback (rtfMRI-nf) is in essence ”closed-loop brain training” ([15]), and allows participants to achieve control of brain activity during fMRI sessions [16, 17] (Fig. 1c). An important principle concerning how strategic thinking may activate predetermined parts of the brain was demonstrated when guided fMRI was used to detect awareness in presumed vegetative patients in 2006 [4, 18] (Fig. 1d). This method, with its high degree of spatial control, is foreshadowed to advance clinical neuroscience [19].

The field of rtfMRI-nf has developed rapidly. In a review from 2018, Thibault et al. claimed that the results were promising, but more research was needed ([7]). A review of rtfMRI-nf on patient populations corroborated the promising results (Tursic et al., 2020), and highlighted the need of larger sample sizes [20]. Moreover, a quantitative meta-analysis of controlled rtfMRI-nf experiments treating psychiatric disorders found large-sized neuronal effects after training and small-sized effects with respect to behavioral outcomes ([21], 2021). The rapid pace of development in this field has made it challenging to establish a common standard. However, a Consensus on the Reporting and Experimental Design of clinical and cognitive-behavioral neurofeedback studies (CRED-nf) checklist and a study design guide have been published to enhance consistency (2020, [22, 23]).

The rtfMRI-nf feedback is displayed through a Brain Computer Interface (BCI), and several BCI options exist, including commercial (Turbo-Brain Voyager, [24, 25]), open-access (OpenNFT, [26]), and cloud-based approaches ([27]). The feedback displayed in such BCIs is the measured and analyzed signal translated to an intuitive and unambiguous output. This may take the form of, for example, a developing graph, a bar/thermometer, clarity of images, and/or a rocket man, depending on the study’s goal and the sample’s participants [28, 16, 29, 30, 31]. The feedback may be calculated based on a region of interest (ROI) ([28, 32, 16, 33, 30]), brain connectivity ([34, 35]), and/or networks [36].

Time has been identified as an important parameter for patient effects in ongoing studies ([37]), and long-lasting effects in connectivity have been observed in several studies [34, 5, 38]. Furthermore, a review examined white matter (WM) plasticity in the adult brain and its potential role in lifelong learning ([39]), and a study has demonstrated - through DTI - how rtfMRI-nf can be used to modify WM structures in corpus callosum [40].

Protocols investigating the effects of biofeedback and neurofeedback have been developed for various populations and conditions. However, the field of fMRI-neurofeedback is still in its early stages ([41]), and the intersection of fMRI-neurofeedback and virtual reality (VR) is even more nascent. Meta-analyses published after this study’s completion suggest that the efficiency of neurofeedback is promising, but protocols remain heterogeneous, and the majority of studies still use EEG-neurofeedback ([42, 43, 44]). Studies have been performed where VR has simulated an MRI experience ([45]), VR has been used to prepare children for MRI ([46]), and studies have used VR during hemoencephalographic ([10]) and MRI sessions ([47]). Recent studies have explored the use of fMRI VR-like stimuli to prepare PTSD patients for VR training ([48]), machine learning of biofeedback to optimize VR exposure therapy ([49]), the combination of biofeedback, VR, and mobile technology to enable home training through sham-feedback ([50]), and NIRS neurofeedback for at-home use by patients ([51]). To the best of the authors’ knowledge, this is the first protocol aiming to explore how targeted use of contextual memory/VR immersion, and home training with sham-feedback may enhance subsequent real-time fMRI neurofeedback sessions.

The aim of this paper is primarily to describe the development and preliminary evaluation of our protocol for real-time fMRI neurofeedback (rtfMRI-nf) and secondarily to explore the potential for behavioral enhancement by implementing systematic contextual memory.

### 2.1. Conditions to treat

We explored the development of a protocol to treat patients with ADHD and Tourette’s Syndrome, replicating the ROIs of [32] and [28] (rIFG and SMA, respectively) in the treatment. By changing the ROIs in the algorithms, the protocol can be tailored to enhance/decrease activity/connectivity in any subregion of the brain.

As studies on neurodevelopmental disorders often target a younger population and SiV specializes in adult patients, we chose to explore the development of this treatment to treat adult patients. Results from neurofeedback studies have shown promise for these diagnoses, and analyses of the effects of stimulants and network deviations for these conditions have highlighted deviations in specific regions that can be targeted by neurofeedback. Furthermore, access to all of the required expertise and instrumentation at OUH/UiO/SiV allowed this exploration. This form of brain training shares parallels with physical training; for example, more training yields larger effects. However, as MRI scanning is both difficult and expensive, we chose to include VR and contextual memory to test if repeated sessions in VR could yield similar effects. If so, this would greatly heighten the applicability of the treatment, as patients could use a VR headset and continue training at home.

**Figure 2:**
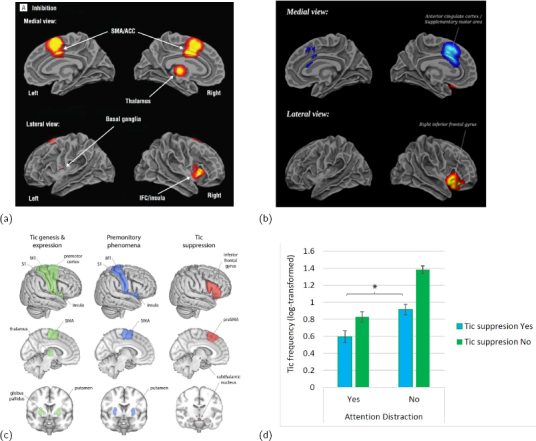
(a) regions exhibiting decreased activity in the brain during inhibition in patients with ADHD includes SMA and rIFG; (b) methylphenidate - a common drug to treat ADHD - stimulates rIFG and decreases activity in SMA; (c) a mapping of regions with deviations correlating with TS symptoms includes SMA and rIFG; (d) conscious tic control and attention may sum to decrease tics in adult TS patients, the two potentially controlled by SMA and rIFG, respectively. Figures (a,b,c,d) from ([53],[54],[55],[56]), respectively.

ADHD is characterized by pervasive and impairing symptoms of inattention, hyperactivity and impulsivity (deviations with respect to (wrt) inhibition, Fig. 2a), and affects around 5% of children and adolescents, and 2.5% of adults worldwide [52]. Patients may benefit from a combination of pharmacological and psychosocial structured treatments, where psychostimulants (e.g. Fig. 2b) are the gold standard for reducing symptoms ([57]); nonstimulants are less effective, and psychosocial treatments are recommended to learn to master the symptoms and deal with them in a more adaptive way [52]. However, since many patients experience little benefit and/or severe side effects from pharmacological agents, there is a need for new treatment options. The evidence for the longterm efficacy of psychostimulants is limited, and brain adaptation may be related to psychostimulants losing their effectiveness over time [57].

Tourette’s Syndrome is a neurological condition appearing in childhood recognized by motor and vocal tics. The earliest and most frequent tics include blinking of the eyes, facial movements, and neck movements [58]. The diagnosis requires multiple motoric tics and at least one vocal tic which have lasted for at least one year [59]. In Norway from 2000 to 2010, 0.43% of children received a diagnosis of TS by the age of 12, and the internationally estimated prevalence of TS in children aged 6 to 15 years is 0.77% [60]. For a sizable number of patients, the condition persists into adulthood, and it is estimated to affect 0.08% of adults [59]. (Regions of the brain associated with TS symptoms are seen in Fig. 2c.) Pharmacotherapy and Habit Reversal Training/Exposure and Response Prevention/Comprehensive Behavioral Intervention for Tics ([61, 62, 63, 64, 65, 66, 67, 68]), are strategies used for treating TS. However, many individuals experience insufficient relief from these options, and there is a pressing need to develop new therapies for adults with TS. A new therapy - for adults - based on attention distraction shows promise [56] (Fig. 2d). Many patients diagnosed with TS have comorbid ADHD, OCD, and/or anxiety. The prevalence of comorbidity is as high as 90%, and these comorbid conditions often have a more significant impact on the Quality of Life of the patients than the tics themselves [59].

### 2.2. Regions of interest

Treatment of ADHD (children) and Tourette’s Syndrome (adolescents) through rtfMRI-nf has been studied previously in [32] and [28], respectively. The ROIs of interest in these studies were rIFG ([32, 31]) and SMA ([28]).

For Tourette’s Syndrome (TS), tics (”unvoluntary” movements, neither voluntary nor involuntary, [72]) may have an origin in SMA; [73] showed that 2 seconds in advance of a tic, SMA was activated in TS patients. For patients with ADHD, a general hypoactiation of rIFG wrt inhibition was found in a meta-analysis ([74], Fig. 2a) and a stimulant (methylphenidate) which relieves the symptoms stimulates rIFG ([54], Fig. 2b).

**Figure 3:**
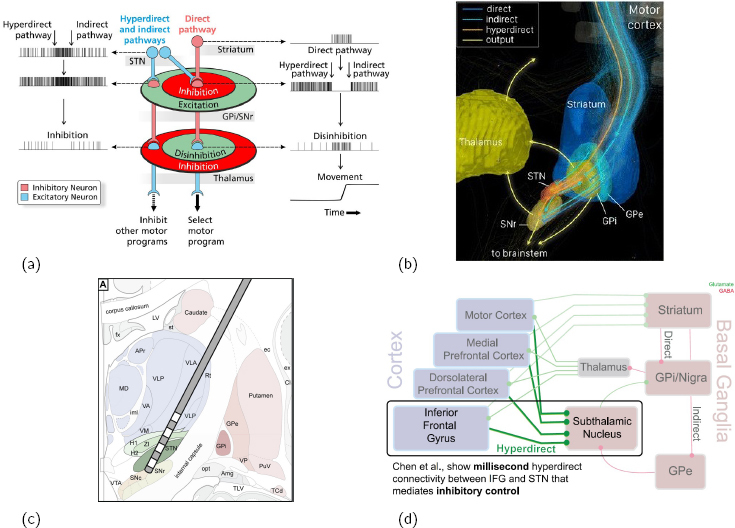
(a) motor control through the tripartite model; (b) how the hyperdirect pathway bypasses the striatum wrt motor inhibition; (c) a common subthalamic nucleus placement of the DBS electrode in Parkinsons Disease patients, stimulating the hyperdirect pathway ; (d) an update of the tripartite model. Figures (a,b,c,d) from ([69],[69],[70],[71]), respectively.

A review on TS highlighted deviations in *corticostriatothalamocortical* (CSTC, [69]) circuits, where the direct, indirect and hyperdirect pathways dictate motor control (Fig. 3a). The same review showed a model of how the hyperdirect pathway bypasses striatum in inhibiting movement control ([69], Fig. 3b). Post-mortem studies have shown that TS patients can have a 50% reduction in GABAergic interneurons in striatum (part of the CSTC, [55]), and a recent PhD study demonstrated how attention distraction could be used as a treatment for adults with TS ([56], Fig. 2d).

A common placement of Deep Brain Stimulation electrodes for treating movement disorders is in subthalamic nucleus (STN) ([70], Fig. 3c). An investigation with high spatial and temporal resolution through field potentials in the human cortex revealed that the hyperdirect pathway between inferior frontal gyrus and STN indeed exists in humans, and that it mediates rapid stopping ([75, 71], Fig. 3d). An investigation using diffusion tensor imaging to map the connections of right inferior frontal gyrus corroborated the connection between rIFG and STN [76].

**Figure 4:**
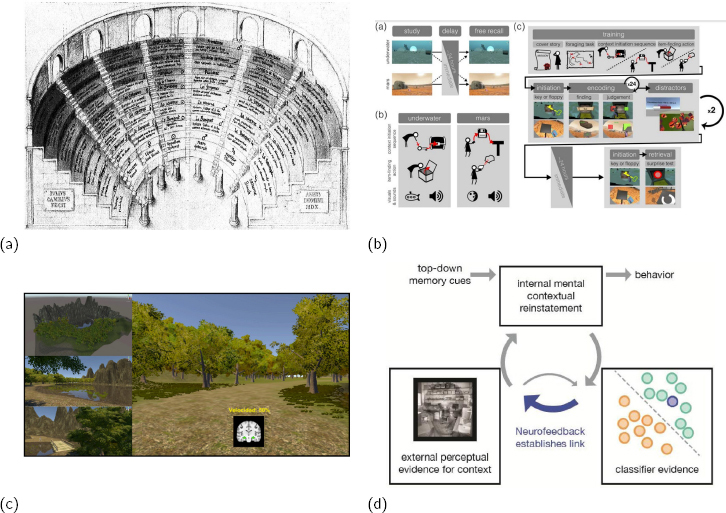
(a) a memory palace used in 1511 AD, providing a virtual context to what was to be remembered; (b) how context in VR was used in deceived participants, indicating how context may aid memory; (c) a VR game used during an rtfMRI-nf run to assess susceptibility for developing Alzheimer’s disease; (d) a hypothesized model for how context reinstatement may aid memory. Figures (a,b,c,d) from ([77],[78],[79],[80]), respectively.

A meta-analysis of fMRI studies on inhibition (and attention) in patients with ADHD found a systematic reduction in activation in both SMA and rIFG concerning inhibition [53]. Based on these results, we aimed to enhance this pathway.

As rIFG may be trained as a consequence of the rtfMRI-nf method ([33]), we chose to provide feedback from SMA and measure resulting effects on both SMA and rIFG. Reinforcing both ROIs could aid both conditions; the primary interest is the relative importance of the ROIs for the benefit of the patients. Recent research has led to the hypothesis that ADHD and TS, and OCD and autism, may actually lie along a impulsivity-compulsivity spectrum [81].

### 2.3. Virtual Reality enhancement

The use of fMRI as a tool for brain training is expensive and time consuming both for the patients and health care providers. In need of a simplified brain training system, we used Virtual Reality (VR) goggles containing a visual experience mimicking their prior rtfMRI-nf training. VR based brain training lacks the feedback from the fMRI-feedback loop and is similar to sham-feedback sessions (which display prerecorded feedback), commonly used as a control in rtfRMI-nf studies ([28, 34, 82, 24], if not for ethical reasons [16, 3]).

Since many factors influence neurofeedback performance ([83, 84]), including psychological aspects ([85]), we aimed to test how episodic memory/VR could be used to enhance the reinstatement of the learning setting for rtfMRI-nf. Context to aid memory has been used since the ancient times (Fig. 4a), and VR induced context was shown to aid memory (Fig. 4b). Neurofeedback through VR has been used to test memory (Fig. 4c), reinstate context to aid memory ([86]), and experiments have been done to induce a mental context through rtfMRI-nf [30]. A model for how context may aid memory has been developed by deBettencourt et al. [80] (Fig. 4d).

We tested if VR simulation could trigger contextual/episodic memory ([87]) in a way that would allow us to extend the brain training, yielding results similar to rtfMRI-nf sessions. Our intention with using VR shares similarities with why participants in [32] were asked to practice daily between sessions of rtfMRI-nf; participants were given a cue-card depicting the feedback and asked to train by remembering the training in the MRI-scanner.

Our VR-based approach parallels the preparation of participants for MRI scans through exposure therapy using mock scanners and VR approaches [88, 46, 45]. Using VR during an MRI scan has been shown to reduce anxiety and improve results [47]. VR used to aid in reinstating context has been shown to enhance memory ([78]), and VR training has been shown to enhance attention in patients with ADHD [89]. Neurofeedback to practice motor imagery was also shown superior to repetitive practice ([90]), and a skill-based VR Cognitive Behavior Therapy has been shown to reduce chronic pain [11].

When exploring the development of this protocol for VR enhanced rtfMRI-nf, we used the open-access software OpenNFT as the rtfMRI-nf platform [26]. For the initial in vitro testing, we used the open-access data in [91] accompanying [26]. Using this platform and this dataset, we collaborated with radiologists/radiographers and translated the in vitro setup to perform continuous classification-based feedback (Case study 3 in [91]). We wrote code/algorithms and developed a gamified feedback design (based on [32] and [28]), and incrementally learned to tune the protocol through pilots. We wrote protocols and code for preprocessing and analysis using Matlab and accompanying toolboxes when needed. Included in the development was the simulation of the rtfMRI-nf experience through VR, the sham-feedback itself was based on screen-recordings of participants, as used in e.g. [28, 33].

## 3. Methods and analysis

The software used includes Matlab and the toolboxes SPM ([92, 93]), CONN, and Anaconda. Checks of MRI-images versus ROIs were done through the free crossplatform software ITK-snap ([94]) and MRICron ([95]).

### Informed consent

Consent was obtained in both verbal and written form. Participants were provided with information about the purpose and procedure of the study in written form, to which they agreed in advance of the initial trial. Further information was given verbally by the first author on each trial day, witnessed by the second author and radiographers. Radiographers ensured participants complied with safety precautions regarding MRI scans in advance of all trials.

### Ethical Approval

The research related to human use has been complied with all relevant national regulations, institutional policies and in accordance with the tenets of the Helsinki Declaration, and has been approved by the authors’ institutional review board or equivalent committee.

### 3.1. Defining the ROIs

As outlined in Sec.2.2, the developmental goal was to provide neurofeedback from SMA and rIFG. The ROI in the rtfMRI-nf procedure is the selected subset of the brain from which the activity is analyzed, feedback is calculated, and then presented to the participant. This study was an ongoing research project to develop both the scientific argumentation of the protocol and the competence to perform it. The different phases of the study thus targeted the given ROIs with increasing precision.

In Pilots 1-4, we used the mask in parietal lobe defined by [26, 91]. For a coarse restriction, WFU_pictatlas ([3, 96]) may be used, and this was how we defined the ROIs during pilots 5 and 6 (rIFG pars triangularis and pars opercularis through the Brodmann Areas 44 and 45 based on [97]). The ROIs were defined with a dilation factor 2 to ensure that the ROI contained the volume across participants. For pilot 7, we used the Julich Probabilistic Maps ([98]); which is probabilistic maps based on averaged/simulated dynamics in the human brain, defined in MNI-space. We chose this atlas as it is the foundation of the Multilevel Human Brain Atlas, is open to all neuroscientists via the Human Brain Project’s research infrastructure EBRAINS, and due to previous experience of one of the researchers in using regions defined through this atlas for neurofeedback. We reinforced SMA (based on e.g. [28, 5, 83, 37, 99, 33]) and rIFG ([32, 97, 54, 100, 101, 33, 56]).

In the EBRAIN database ([102]) we used the *JULICH_BRAIN_CYTOARCHITECTONIC_MAPS_2_9_MNI152_2009C_NONL_ASYM* to define the ROIs. The 3D reconstructed histological datasets in [102] are transferred into two reference spaces, the single subject MNI-Colin27 space, and non-linearly transformed into ICBM2009casym space; the latter being a compromise between the anatomically detailed MNI-Colin27 and the more generic but smoother MNI305 template [98]. Through the accompanying text-file one finds that SMA (in both hemispheres) is defined through areas 135 and 136, and that rIFG is defined through area 120 and 122.

As both rIFG and SMA are defined through two subregions, we combined these subsections using temporary variables and performed voxel-by-voxel summation in a custom-written Matlab script. For voxels where the ROIs to be combined were overlapping, we used the maximum value.

### 3.2. Calculating the feedback

For the real-time fMRI calculations, we used OpenNFT [26, 91]. The rationale for choosing OpenNFT over e.g. Turbo Brain Voyager was financial, OpenNFT is open-access, which also induces easier communication and replication of the protocol by other scientists. The OpenNFT platform is a BCI that takes the ROIs to be investigated as input and analyzes the activity of this/these ROI(s) during the experiment. We wrote a gamified feedback where the output from OpenNFT was transferred into a game where a rocket was flying through space during activation-blocks (resembling [32]), and a submarine was sinking during deactivation-blocks, with a goal to obtain the maximum, summed score. As the output from Open-NFT was single number between 0 and 1, we reversed the feedback by using 1-OpenNFT feedback during downregulation blocks (thus, no rest-periods, [28]); maximum BOLD signaling in the chosen ROI then effectively yields a minimum score in the feedback during these periods.

Reinforcing both activation and deactivation was chosen to enhance conscious control of the ROI ([28, 5]). The feedback was mirrored onto a LCD screen behind the MRI-scanner, and the participant in the scanner only saw the rocket/submarine, and the updating score. In addition to the rtfMRI-nf sessions and a sMRI, we included a 12-volume pre-fMRI sequence to be used in adapting the ROI to the fMRI-space of the person in the scanner.

In our exploratory development we initially developed a procedure in line with the setup of OpenNFT, a block design with task and rest periods tunable through an OpenNFT configuration file ([32]). In this configuration file, output during rest periods equals zero. As we wanted to purposefully enhance deactivation of the ROI during rest periods ([28]) in our protocol, we chose to change the paradigm and developed a “block-design” consisting of 2 blocks. (The results from pilots 1-4 and pilots 5-7 are thus not comparable.) The rest block contained only the initial 10 volumes (for saturating the magnetic field), and the remaining 200 volumes constituated the task block. This task block was further subdivided in activation and deactivation periods, which were not visible in OpenNFT, but were presented in the feedback to the participant in the scanner.

The blocks were a compromise between [28, 32, 26], where we recorded 210 fMRI volumes (as in [26, 91]), designed a gamified feedback based on [32], and adapted the block length from [28] (20 volumes) to 18 to create an equal length of all blocks according to our repetition time (TR, 2.15 seconds).

### 3.3. Transforming from MNI to personalized fMRI-space

**Figure 5:**
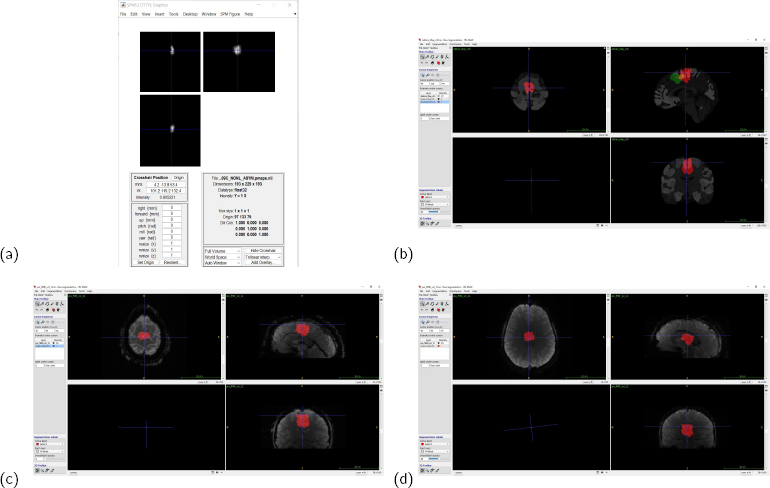
Examples from the ROI extraction and later adaptation. (a) SMA in the right hemisphere as directly shown through the SPM display code shown earlier; (b) the combined SMA in MNI space shown in green, and SMA realigned according to the pre-fMRI volume of a person having gone through the SPM adaptation algorithm (in red), the required shift according to fMRI-space is evident; (c) the same shifted ROI from (b) shown according to the fMRI volume of the person to be trained; (d) the same ROI from (b-c) overlaid the pre-fMRI of a different person having gone through the rtfMRI-nf, the need for realignment is evident.

The Julich Brain Atlas ROIs in Sec.3.1. are defined in MNI-space. To be able to track the ROI precisely during the rtfMRI-nf, a conversion of the ROI from MNI-space/coordinates to fMRI-space is required. We wrote an SPM Batch algorithm to adapt and convert the MNI-space-defined ROI. This conversion was done through a combination of an sMRI volume and an fMRI volume recorded first during the rtfMRI-nf training. The purpose of this conversion was to accurately target the specific region in the brain of the person in the scanner. Details of extracting the ROIs and of the SPM Batch process is described in SI.1-2; examples are shown in Fig. 5.

### 3.4. Preparing participants

Participants were given a general introduction to the principles of the procedure in advance of the experiments. The way this exploratory protocol is designed is with the goal of keeping the invasiveness to the participants to a minimum; therefore, our procedure did not require any additional information other than what was recorded during the rtfMRI-nf training sessions. Developing an objective, highly standardized protocol may circumvent the need for a localizer scan (as in e.g. [28, 103, 24]). The ROIs targeted were chosen due to anatomical deviations correlating with the diagnoses. Therefore, given that the patient is correctly diagnosed with the condition, they would benefit from our selective brain training when targeting the intended anatomical position. We chose to use a method to translate the anatomical MNI-space data of the regions to the participant fMRI-space to ensure individual adjustment (SI.2). This also removes the need for prerecorded fMRI data to be used, for example, in machine learning, to enhance precision (as in [104]). With regard to safety, in collaboration with professional radiographers, the preparation to and conduction of the rtfMRI-nf was similar to a normal MRI-session. Recruitment, information and preparation of the participants are described in SI.3.

While performing the rtfMRI-nf, participants in the MRI-scanner saw an animation of a spaceship flying upwards. In activation blocks, the speed of the rocket was dictated by the simultaneous activation of regions within their brain. Activation blocks were separated by periods of rest, during which no feedback was given. Starting from pilot 5, we modified the paradigm by replacing the resting periods with periods where active deactivation was reinforced. The VR simulation included a walk through the hospital toward the MRI-scanner, followed by a sham-feedback session based on a random sampling of screen recordings of earlier rtfMRI-nf sessions.

### 3.5. MRI-scanner and parameters

The experiments were performed at Oslo University Hospital, on a Siemens Magnetom Prisma 3T whole body MR scanner ([28, 91, 92]). For the fully developed protocol, each session consisted of the following: 1) a 12-volume pre-fMRI resting-state scan, 2) a T1-weighted sMRI scan, and 3) three rounds of rtfMRI-nf. The first two scans were used in a Matlab/SPM batch fMRI-space adaptation algorithm. The 12 volume pre-fMRI scans were recorded using a T2*-weighted EPI sequence TR/TE = 2150/28 ms, flip angle = 74°, 35 × 2.5 mm slices with a 0.6 mm slice gap, 22 cm FoV, and voxel size = 2.2 × 2.2 × 2.5 mm^3^. The T1 weighted scans were recorded with TR (repetition time)/TE (echo time) = 1.900/3.16*ms*, flip angle = 9°, 192 × 1 mm slices, matrix size 256 × 256, 256 mm FoV, voxel size = 1.0 × 1.0 × 1.0 mm^3^. The following three rounds of rtfMRI-nf were recorded with the same parameters as the initial 12 volume pre-fMRI resting state scan.

### 3.6. Running the rtfMRI-nf

Our rtfMRI-nf protocol required the synchronization of the laptop performing the real-time analyses with the MRI scanner, enabling the software to expect and wait for incoming fMRI volumes. The repetition time (TR) of the MR-scan was set such that an entire loop of MRI-transfer, MRI-analysis/feedback calculation, and feedback display were conducted within each TR. (OpenNFT has additional safety checks to ensure analyzing the correct sequence.) The setup led to the raw MRI image being sent to a shared folder (shared between the MRI scanner and the laptop), whereas another program renamed and transferred the MRI image to a new folder. In this folder, OpenNFT expected the images to be analyzed to appear and analyzed all incoming files. OpenNFT was configured to analyze the BOLD data for specific ROIs within the fMRI volumes, and the calculated feedback was subsequently sent to our gamified feedback display, which is mirrored onto the LCD screen at the back of the MRI scanner. This loop was repeated for each fMRI-volume. We used OpenNFT version number 1.0.0rc0 and matlabengineforpython 2021a. The laptop-fMRI synchronization was based on [105]. The setup is further explained in SI.4.

Radiographers translated our experimental setup and rtfMRI-nf block-design and created a MRI-template. Our design included a 12-volume pre-fMRI resting state scan - with the same parameters as the later rtfMRI-nf sessions -, then subsequently a T1 weighted sMRI, in advance of three rounds of rtfMRI-nf. A 12-volume pre-fMRI was used to ensure that the magnetic field was homogenized/saturated at the 12th volume. This volume was then converted from dcm to nii format. The sMRI was first converted to axial slices, then converted to nii format, and subsequently combined to a stack through FIJI/ImageJ. The converted 12th volume pre-fMRI and sMRI files were then processed via the normalization/MNI-fMRI-space SPM Batch algorithm described in SI.2.

After the completion of the SPM Batch algorithm, the output files underwent further processing through another Matlab algorithm to create two masks: one weighted mask that was filtered to remove residuals (post-filtering/smoothing, with the deletion of voxel values under 0.1 and above 1.0), and a binarized mask. The binarized mask involved first deleting voxel values below 0.1, followed by setting all non-zero voxel values to 1.0.

### 3.7. VR home training

To assess if we could utilize VR to enhance the efficiency of the treatment, we developed a VR platform with a sham replication of the rtfMRI-nf experience to be used for home training. The sham feedback was created through screen recordings of rounds of rtfMRI-nf run in vitro on data collected in earlier pilots. To aid the reinstatement of the mental context surrounding the experience, the VR experience included a walk through the hospital toward the MR-scanner. The MR-scanner noise was also added during the sham-feedback. The VR experience was made as simple as possible, such that the participants only had to press “Play” after mounting the VR-headset (SI.5).

### 3.8. Analyzing rtfMRI-nf

Analysis of the results was conducted using the Matlab SPM-based toolboxes CONN and REX ([106, 107]) for the analysis of the connectivity and activity of the ROIs, respectively. Details of this analysis are described in SI.6. We chose to register each round for each participant as an individual subject to prevent the automatic averaging of participants. For instance, a participant with two separate sessions, each containing three rounds of neurofeedback, resulted in six different sets of fMRI scans, but only one T1-weighted sMRI was imported.

To ease replication and enhance standardization, we conducted the analysis following the procedures suggested by CONN, only actively selecting the additional REX output. Through the standardized 4-step analysis setup, the CONN GUI culminates in an interface where one can e.g. choose to selectively investigate how the connectivity between two isolated ROIs - predefined from a Harvard- Oxford brain atlas - changed during the fMRI scans in the study. Through the extra REX output, the analysis also exported additional files (interrogated through the Matlab toolbox REX) where the activity pattern for each isolated subregion could be displayed.

### 3.9 Performing the protocol

**Figure 6:**
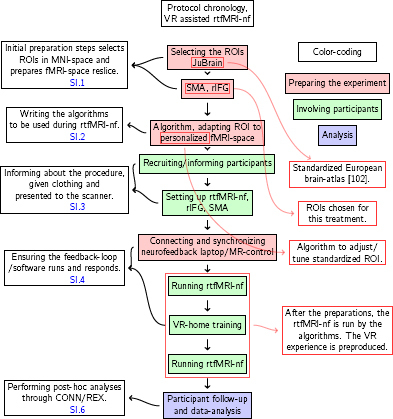
Abbreviations: fMRI=functional magnetic resonance imaging, rIFG=right inferior frontal gyrus, rtfMRI-nf=real time fMRI neurofeedback, ROI=region of interest, VR=virtual reality

In this section, an overview of the protocol is given in Fig. 6, and the details of the steps are described in the supporting information (SI). Links to respective sections describing the steps are included in the flow diagram.

### 3.10 Pilots/representative results

**Figure 7:**
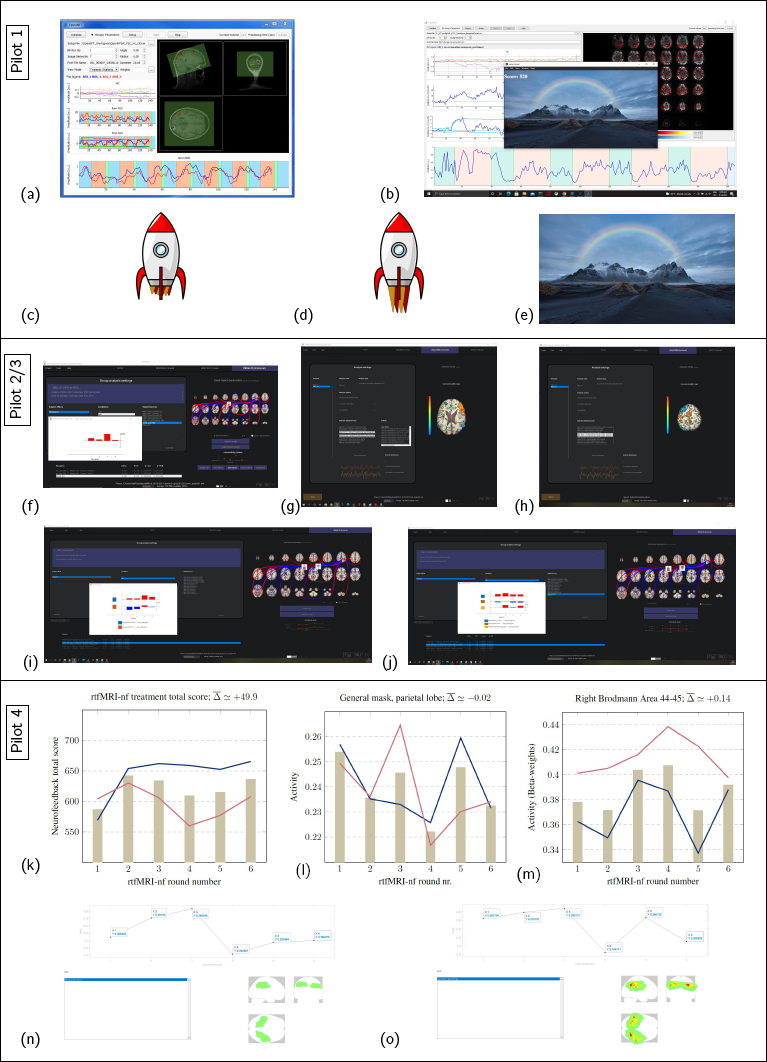
The pilots and the results are described in the Pilots/representative results section, Sec.3.10.

**Figure 8:**
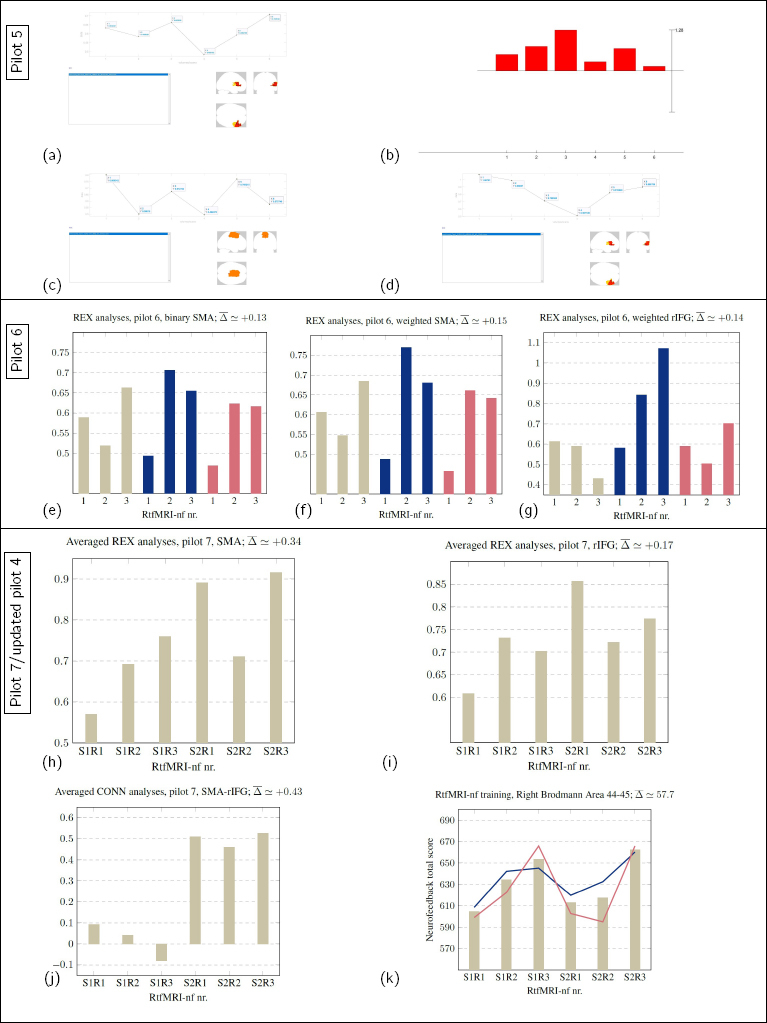
The pilots and the results are described in the Pilots/representative results section, Sec.3.10.

In this study, we explored the development of a protocol to conduct rtfMRI-nf. Additionally, we performed preliminary testing to investigate how VR may potentially enhance the treatment through sham feedback home-training. We performed pilot testing throughout the exploration of the different stages of the procedure. Pilot 1 was performed purely in vitro, pilots 2 and 3 were conducted on a singlesubject each, pilot 4 involved 10 volunteers, pilot 5 involved 2 volunteers, pilot 6 involved 3 volunteers, and pilot 7 involved 2 volunteers. Representative results from the 7 pilots are displayed in Fig. 7 and Fig. 8.

First we made the platform work in vitro, and Open-NFT as seen in [26] is displayed Fig. 7a. Fig. 7b shows the end display after an early pilot using our adjusted software; the center window is the feedback display, and the end score in this round was 520. In Fig. 7c-d, the rocket, which represents how the feedback was presented, is displayed in its minimum speed (Fig. 7c) and maximum speed (Fig. 7d), and the image used for relaxation blocks is shown in Fig. 7e. We initially used a block design with activation vs. rest ([32]), but later decided to reinforce activation and deactivation ([28]).

Connectivity-analyses of the MRI-Images from the rtfMRI-nf pilots 2 and 3 (tested on the first author, 2 sessions, 1x3 and 1x1rounds) through the Matlab toolbox CONN is shown in Fig. 7f-j. Furthermore, we tested whether the connectivity of our initial rIFG mask to the mask given feedback changed during the training, as indicated in the three sequential rounds (Fig. 7f). In Fig. 7g-h sections from the CONN definition of rIFG and SMA is shown, respectively. In Fig. 7i-j connectivity analyses from the CONN definitions of rIFG (both the pars triangularis and pars opercularis) are shown. Fig. 7j includes a refined definition of the combination of rIFG. Of primary interest in Fig. 7i-j is the indication of the effect of time and sequential training. As these were results from rtfMRI-nf of the same participant spread across two times, the heightened activity level in the fourth column (with respect to the first) may indicate that the participant learned/the brain matured from session 1 to session 2.

In pilot 4 we conducted a pilot of 10 volunteers, the results from which are shown in Fig. 7k-o. In Fig. 7k, the average total score for each of the participants are shown through the 6 rounds of rtfMRI-nf, divided into two sessions, separated by 1 week. In Fig. 7l-m the averaged developing activity level through REX analyses of the binarized mask given feedback and rIFG is shown. The mask given feedback (Fig. 7l) did not display an increasing trend, which was in contrast to the activity in rIFG (Fig. 7m), which indicated an increased activity level from start to finish. In Fig. 7n-o example images are shown from REX analyses of the activity in the binarized mask and the weighted mask, respectively, in the parietal lobe for one of the participants in the 4. pilot. Limitations of this pilot include e.g. the definition of the ROI masks, and the use of fMRI-space adapted masks to a different MRI-scanner and different brains.

For pilot 5, we tested redefining the ROI masks given feedback on two volunteers and created a mask of rIFG for the first volunteer and a mask of SMA for the second volunteer (Fig. 8a-d). We also altered the block design of the neurofeedback, replicating the method described in [32] for rtfMRI-nf of the first volunteer and the method described in [28] for rtfMRI-nf of the second volunteer. The masks were created in WFU_pickatlas and output in MNI-space, but the reslicing of the masks was done based on earlier pilots. The first volunteer did show a small increase in the activity level of rIFG given feedback (Fig. 8a), but the connectivity between rIFG and SMA did not increase (Fig. 8b). The second volunteer did not show an increase in activity in either SMA or rIFG.

In pilot 6 we tested the use of support vector machine learning to enhance the efficiency of the neurofeedback protocol. Based on [26, 91], we used the PRoNTo toolbox ([108]) to test adapting the mask given feedback through the brain training, replicating [109]. Due to the time required for updating the masks, we only updated the mask once (during the sMRI), and did one session of rtfMRI-nf on three healthy volunteers, all training SMA. REX results from these pilots are shown in Fig. 8e-g. The results show that the activity in the binarized SMA increased from the start to the finish for all three volunteers (Fig. 8e). The activity also increased in the weighted SMA mask being PRoNTO updated (Fig. 8f), and similarly, for two of the three volunteers, the activity in rIFG also increased through the training (Fig. 8g).

In pilot 7 sham feedback was tested, both in the MRI-scanner, and during the VR-home training. Due to an unforeseen event, we were forced to conduct sham feedback on both volunteers in session 1, and the VR-home training therefore lost a lot of its purpose as we have no reference to which to evaluate the performance of the volunteers. Analyses of the neurofeedback training sessions through REX and CONN are shown in Fig. 8h-k. In this pilot the MRI-protocol was updated, and additional software was written to include adaptation of the mask to the fMRI-space of the volunteer in the MRI-scanner. In Fig. 8h and Fig. 8i the averaged activity of SMA and rIFG through the sessions are plotted, respectively; evident from these plots is that the activity of both the ROIs for the two volunteers increased through the brain training. Also of interest is the fact that there is a marked activity increase from session 1 to session 2. In Fig. 8j the connectivity between SMA and rIFG is plotted. Also evident from this plot is the general increase in the connectivity between the two ROIs. This increased correlation of activity may indicate that conscious use of SMA, reacting/adapting to the neurofeedback, may enhance the activity of rIFG. Feedback from the volunteers revealed that the VR-platform need further development. The similarity of each round of the VR-home training was less inspirational than the neurofeedback training at the hospital, resulting in them not performing much home-training.

In Fig. 8k results from an *in vitro* rerun of data from pilot 4 is shown, where feedback was shifted from the parietal mask from [91] to personalized rIFG. The result from this analysis also indicates that rIFG is enhanced through the rtfMRI-nf sessions, in accordance with [33].

## 4. Discussion

This article describes the phases of exploratory development and preliminary evaluation of a protocol for real-time fMRI neurofeedback (rtfMRI-nf), as well as the potential inclusion of enhancement using stimuli from Virtual Reality (VR), which could be relevant in the treatment of patients with ADHD and TS.

We applied OpenNFT as a basis for the protocol, and wrote Anaconda, MATLAB, and SPM algorithms to enhance the precision of the protocol. The testing of the protocol yielded positive preliminary results. One of the strengths of this protocol is that it is easily generalizable to any given region of the brain, and to e.g. different block-designs and feedback paradigms.

OpenNFT was experienced accessible, and tuning this software to our experimental needs was relatively easy. As the output from OpenNFT is provided in sequential numbers in Anaconda, creating a feedback program that responds to the OpenNFT output allowed for an accessible feedback display. Updates and enhancements for Open-NFT are continuously in development, as demonstrated in [110].

The use of the standardized Julich Brain Map provided a more precise location for the ROI to be reinforced compared to the use of WFU_pickatlas. Additionally, a foundation in updated structural segmentation enables further refinement of the brain training, allowing for more precise adjustments.

Developing the VR-platform to be used for the hometraining was completed in a purely functional sense, but we found that the immersive quality of the developed VR-platform needs to be updated to increase participant motivation for conducting the home-training. Options for enhancing the usability of the VR-platform could be to e.g. include a VR-simulation of the MRI-scanner where the participant could interact more with the room/scanner/ consciously choose to enter the scanner, the neurofeedback could be made more visually/intellectually stimulating, the walk through the hospital could be filmed with a 360 degree camera to induce a stronger sense of presence at the hospital and/or this step could be deleted in general.

Without feedback from the brain when using the VR-headset, this neurofeedback setup will need further development for adult use. The intuitive, gamified feedback rapidly became predictable when the participants knew that the feedback was fake (despite 9 sham-feedback sessions were programmed and randomly selected in the VR-training). The platform might have had a stronger effect on children (although the ethical considerations are challenging), but for the VR platform to achieve the intended effect, more focus must be placed on replicating the experience. Alternatively, a different form of feedback, such as functional near-infrared spectroscopy (fNIRS), could be considered. The use of fNIRS is a far more flexible approach and enables recording and providing feedback based on a similar haemodynamic response as in the fMRI, without the need of the restrictive MRI-scanner, but is restricted to the top centimeters of the cortex [111]. However, the fNIRS approach has been compared and validated for investigating SMA ([112, 113, 114]) and IFG ([115]). The use of sequential rtfMRI and rt-fNIRS feedback for stroke rehabilitation was found to significantly improve motor function ([116, 117]). Using fNIRS neurofeedback for treating children with ADHD has also recently been done, showing promising results, especially concerning attention deficit symptoms [118]. An additional option is to replace VR-based home training with transcranial direct current stimulation (tDCS)-based home training, which has been recently implemented ([119]). In this approach, adult participants stimulated their right dorsolateral prefrontal cortex 28 times (once every day) over four weeks, resulting in increased attention. Both longevity and consistency of the study and age of the participants may have been important, as tDCS stimulation in [57] did not yield any clinical change. [57] used tDCS over 15 consecutive weekdays (excluding weekends) targeting rIFG in children, and participants in [119] did not show any changes after 14 stimulations over two weeks. However, unlike rfMRI-nf and rtfNIRS-nf, tDCS is passive stimulation.

The developed algorithm for the MNI- to fMRI-space adaptation resulted in the two sequential sessions more accurately reinforcing the same ROIs. The lack of this in the earlier pilots draws into question if anything other than purely mechanical procedural lessons can be learned from the earlier results. Visualizing/comparing the ROIs given feedback after the fMRI-space adaptation, and comparing the overlap of the ROIs revealed large discrepancies. Hence, this is an essential step, and may also partly explain why the volunteers showed a large variation in achieved feedback control, and also partly why thought strategies which functioned well in session 1 did work not in session 2.

The use of support vector machine (SVM) learning to enhance the efficiency of the neurofeedback was not tested sufficiently to allow inferences to be drawn, but the PRoNTo software was tested in a preliminary pilot, and the resulting weighted voxels output from the analysis did lead to an overall increase in the neurofeedback for the participants conducting the one session rtfMRI-nf. This might be related to the output from the SVM analysis to a larger extent reflecting how the person in the scanner was using the brain. That is, despite the original mask given feedback was not adapted to the fMRI-space of the person, performing a SVM analysis on the first round of rtfMRI-nf may yield a new mask which more accurately reflects how the person used the brain in round 1 when calculating the feedback for the subsequent rounds 2 and 3. This line of thought could be given more importance in subsequent experiments.

The developed protocol avoided the use of a functional localizer task (regarded a weakness of the protocol in [120]), but instead relies on the study focusing on a predetermined area of brain. This setup is therefore applicable for studies e.g. focusing on strengthening/weakening regions shown abnormal in mental disorders, replicating/simulating the effects of drugs through mental training, or for rehabilitation of patients with lesions in the brain, caused by e.g. strokes or surgery in the brain. Easy tuning of the ROI given feedback through the Julich Brain Map and subsequent algorithms allows several patient conditions to be treated, and creation of control ROIs (e.g. controlling though the anti-correlated ROI, [32])is equally simple. The developed protocol also allows for easy manipulation of e.g. the block-design, feedback display, and transfer-runs/sham-feedback.

Through our pilot experiments, we have discovered that the VR simulation used for home training needs to be more similar to the rtfMRI-nf in order to achieve the desired effect regarding how sequential rtfMRI-nf and simulated VR sham feedback could enhance treatment effects. In future experiments, we will replace the passive VR home training between sessions of rtfMRI-nf with active fNIRS feedback (similar to [116, 117]), providing participants with continuous active feedback throughout the treatment.

## 5. Ethics and dissemination

All procedures were approved by the Norwegian Regional Ethics Committee (REK). The volunteers provided their written informed consent to participate in the study. All rtfMRI-nf sessions were supervised by professional doctors and radiologists. This study is a part of a PhD project that will be publicly disseminated.

### Expected Results

Through implementing this targeted brain training on patients, we anticipate being able to measure increases in SMA and rIFG BOLD responses through fMRI analysis. We anticipate that these same increases will be paralleled by enhanced connectivity and activity, as demonstrated through MATLAB analyses using CONN and REX, respectively.

The extended implications of increased conscious control over these brain regions for patients with these diagnoses may reduce the severity of their conditions. However, to demonstrate this, long-term follow-up studies implementing the completed protocol on patient populations are necessary.

### Limitations

During the development, there were no blinding or other controls for biases. All the volunteers were recruited through ad hoc processes; when pilots were to be arranged and the MRI scanner was available, volunteers were contacted through personal networks of the employees at Nordic Neurotech. Therefore, there was no systematic control of the personal characteristics, homogeneity, or representativeness of the samples in the different pilots. But as e.g. different ROIs, different block-designs, and different feedback paradigms were utilized through the project, comparison between pilots can not in any regard be justified. Developing understanding of the software and instrumentation throughout the project also adds another variable dimension.

## 6. Conclusion

The developed protocol was shown to be operational and may be used to conduct rtfMRI-nf on all regions/ROIs of the brain; sham-feedback experiments are required to verify the function of the protocol. The setup describes the procedure for conducting rtfMRI-nf of SMA and rIFG, but exchanging these ROIs with any others can easily be done through substituting the MNI-space ROIs extracted initially (from EBRAINS or other sources) in advance of any processing. By changing the ROI, the strategies advised to the participants must also be changed (or one may choose to not give any strategies), as the strategies applied will induce selective brain activation for ROI-based feedback. Thus, this setup can therefore be used for any kind of BOLD-based regional brain training. We were able to show a trend toward increasing control of a ROI (early pilot), and developments through the project refined the protocol (e.g. enhanced the precision of both the brain training and the analyses, and improved the VR-replication), but we did not reach any conclusive results. We recommend researchers using this protocol to pay attention to potential alterations in the activity of rIFG regardless of the ROI given feedback.

## Supporting Information

For the steps shown in the flow diagram (Fig.6) the developed procedure is conducted as follows:

1. The ROI to be reinforced is first selected through the Julich Probabilistic Maps (JuBrain) [98], which outputs volume coordinates are given in MNI-space. The ROIs we reinforced through the rtfMRI-nf were SMA and rIFG, justified in Sec:2.2..
  (a) The extraction and further tuning is done in MATLAB and SPM.
  (b) The accompanying Julich Brain Atlas text file defines the ROIs.
  (c) One may visualize the ROI to be extracted through the code: *spm_image(‘Display’,julich_brain_colin27_v2_9(136))*;, which displays the probabilistic SMA in the right hemisphere in SPM.
  (d) One then defines the subvolumes of the ROIs to be investigated (e.g. *SMA_right=spm_read_vols(julich_brain_colin27_v2_9(136))*;)
  (e) To combine subregions, we wrote a custom MATLAB script. For voxels where the subcomponents to be combined overlapped, the maximum value was chosen.
  (f) To write out the ROI-file the *niftiwrite*-command was used. E.g. niftiwrite(SMA_right,’SMA_right.nii’) writes out SMA of the left hemisphere; the accompanying information for this file will be in MNI-space.
  (g) In preparation for the MNI-space to fMRI-space conversion, an SPM reslicing of the MNI file to an example fMRI volume, using specific parameters for real-time fMRI neurofeedback, was performed. This is necessary because the SPM Batch algorithm requires the ROI file to have the same dimensions and voxel size.
2. The procedure outlined above can be used to create probabilistic maps for any part of the brain; however, the voxel coordinates are in MNI space. To transform the ROIs from MNI-space to fMRI-space, we developed a specialized four-step SPM Batch code. This algorithm predisposes that both a T1 volume and a single fMRI volume have been pre-recorded. The SPM Batch steps included:
  (a) A *coregistration* step: the pre-fMRI volume is used as a reference to which the MNI space ROI is to be coregistered, and the T1 image is “jiggled to best match the reference”.
  (b) An “*Old segment*” step: matching the parameters in the rtfMRI-nf to be performed, with additional pre-processing/filtering steps.
  (c) An “*Old Normalize: Write*” step: the normalized MNI-ROI files to be realigned are input, and the normalized files are output.
  (d) And a “*Realign: Reslice*” step: the files from the former step are further resliced to match the pre-fMRI volume, ensuring that the MNI-ROIs have been normalized, realigned and resliced to the correct MRI-parameters, in the individual’s correct fMRI-space of the brain in the scanner, in advance of the rtfMRI-nf.
  (e) A custom Matlab-script was written to filter the files, removing non-numerical voxels, which the SPM fMRI-space adapted files were subsequently processed through.
3. The participants are recruited and informed through mail about the procedure and how the brain training is conduced. Included in this main (and later interview) are questions about contraindications for MRI, e.g. strong claustrophobia or pacemakers/implanted metal.
  (a) The participant is given a quick introduction to the physics and brain BOLD mechanisms recorded and given output through rtfMRI-nf, and special note is given tothe ROI to be given feedback in this session. Despite disagreement in the literature concerning if participants are to be provided with thought strategies/instructions (e.g. [28, 32] disagrees, [83] found that reward may be important), we gave the participants example strategies as part of explaining the function of SMA. In a review from 2021 88.2% of rtfMRI-nf studies provides instructions [21].
  (b) But in addition to the recommended instructions, all participants were encouraged to try different strategies, as no two humans are alike, and hobbies/interests is also a part of determining how explicit one manages to visualize the thought in the brain.
  (c) The participants were also informed about the inherent time delay in fMRI due to the hemodynamic response function, and, given the variations in the literature, we explained that this delay is approximately 6 seconds. Therefore, it was emphasized that participants should try each strategy for a minimum of 6 seconds before attempting a different one.
  (d) The participant is provided with MRI-compatible clothing, and a radiographer assists them in entering the scanner.
  (e) Contact is maintained with the participant, and the visibility of the LCD screen is verified before initiating the rtfMRI-nf session.
  (f) After the rounds of rtfMRI-nf, the person is interviewed again, with the main question revolving around which thought strategies worked best during the rounds of rtfMRI-nf.
  (g) When the participant returns for the second session, the procedure is repeated.
4. *Connecting and synchronizing the neurofeedback laptop and the MRI-scanner.*
  Our experimental design includes 1 T1-weighted sMRI, 1 12-volume pre-fMRI scan, and then 3 rounds of rtfMRI-nf. The first two scans are in preparation of the rtfMRI-nf, yielding a two-step process. *Synchronization*:
  (a) Neurofeedback laptop:
    - The neurofeedback laptop is connected to the MRI-scanner through a Network-switch.
    - The local MRI folder on the neurofeedback laptop is shared to a private network.
  (b) MRI-computer:
    - An administrator access is opened on the MRI-computer.
    - In “tools” one selects “Map network drive”.
    - One then selects a drive and folder, and uses the username and password of the scanner.
    - To write out the DICOM files in real-time, for Siemens scanners (which we use), the *ideacmdtool* and the steps in [105] is followed; we documented the settings in advance of changing them to verify the later resetting.
  (c) Neurofeedback laptop
    - *OpenNFT* : an anaconda prompt is opened, and the codes “*conda activate open-nft_venv”, and *“opennft_console*” opens the OpenNFT platform.*
    - In the OpenNFT GUI, “Initiate” is selected, and the parameters of the experiment are verified in “Review Parameters”, such as the configuration file with the block design, the ROIs/masks to be reinforced/subdued, and that the experiment is not set to “Offline”.
    - *Feedback display* : a new Anaconda prompt is opened, and the code “*cd C : \Users\(…)\rocketwsub*”, and “*npm start*” is input, which initiates our program for the feedback display.
    - *Transfer function* : a new Anaconda prompt is opened, and “*cd C : \Users\(…)\watch2*”, and “*npm start*”, which initiates the function which will transfer the incoming images from the shared folder to the local OpenNFT folder where the analyses takes place.
    - A phantom scan may be performed to verify that the synchronization is established.
  (d) *T1 weighted sMRI and pre-fMRI scan*
    - The MRI-experimental design is initiated, and the 12 volume pre-fMRI scan and the T1-weighted sMRI (with a 160 slice axial conversion) is conducted, and the files are transferred from the MRI-computer to the synchronized neurofeedback laptop.
    - The 12th volume of the pre-fMRI scan and the T1 weighted sMRI stack is input into the SPM Batch function to calculate/transfer the ROI from general MNI-space to the personalized fMRI-space of the participant in the scanner.
    - The updated ROIs (also filtered, and subdivided into a weighted and binarized mask) are transferred to the OpenNFT ROI-folder.
  (e) Starting rtfMRI-nf
    - In the OpenNFT GUI, select “*Setup*”, then “*Start*”.
5. VR home training
  The purpose of the VR home training is to make this activity easy for participants to perform, and help them recall the training experience/thought strategies. Therefore, the setup was designed to be as intuitive as possible, and the following steps describe how to use the training.
  (a) Mount the VR headset, and turn it on.
  (b) In the VR display, select “VR home training”.
6. Analyzing the Data through CONN/REX:
  (a) After opening CONN and creating a project, basic information is input, including: the number of subjects, the number of sessions or runs, Repetition Time (in seconds), and Acquisition type: “continuous”. To account for two rtfMRI-nf sessions, each consisting of 3 rounds, with a single participant, we entered the number of participants as 6 and the number of rounds as 1. This configuration avoids averaging between the trials and allows us to track developments from one trial to the next.
  (b) Since all the subjects are, in fact, the same individual (per analysis), we used the same T1 scan as the structural data.
  (c) For the functional data, each round of rtfMRI-nf is first converted to nii files through the *dcm2niix* program, and each round is input in the correct sequence.
  (d) For the ROI, the CONN atlas expects the MNI coordinates, such that the masks input here are the masks directly output from the EBRAINS MATLAB extraction. Inputting the fMRI-space converted masks used in the rtfMRI-nf causes erroneous tracking.
  (e) One then defines the conditions and converts the initiation of each block in the block design into seconds through the TR, and remembering to cut the initial 5 volumes from the first rest period as these volumes are used to homogenize the magnetic field in the MRI-scanner.
  (f) We did neither use any 1-level nor 2nd-level covariates.
  (g) For the “Options” we chose to tick off “Create ROI-extraction REX-files”, which creates individual REX files for the ROIs/masks one include in the former section.
  (h) The standard “Preprocessing” is done, then “Done” is chosen.
  (i) The standard “Denoising” is conducted.
  (j) The standard “Analyses (1st-level)” is conducted.
  (k) For the 2nd-level connectivity analyses, we are using a “ROI-to-ROI” group analysis setting, and choosing “Group analysis results: individual ROIs” to be able to isolate how the connectivity between individual ROIs change through the rounds of rtfMRI-nf.
  (l) The protocol above also creates REX files output in the CONN folder where the project was created (*…″project″/results/firstlevel/SBC_0_1*). These files can be analyzed in the REX MATLAB toolbox by typing “rex” in MATLAB. (In the “list_condition.txt” and “list_sources.txt” one may track which files to include in which analyses.) In the REX GUI one navigates to the REX output folder, and includes the ROI from “which person” (that is, the sequence of rounds) in which condition one wished to analyze. By maintaining the ROI and condition one may see how the BOLD for the ROI develops through the training (and thus over time).
